# Current Insight into the Role of IL-35 and Its Potential Involvement in the Pathogenesis and Therapy of Atopic Dermatitis

**DOI:** 10.3390/ijms232415709

**Published:** 2022-12-11

**Authors:** Weronika Zysk, Jolanta Gleń, Magdalena Trzeciak

**Affiliations:** 1 Dermatological Students Scientific Association, Department of Dermatology, Venereology and Allergology, Faculty of Medicine, Medical University of Gdansk, 80-214 Gdańsk, Poland; 2 Department of Dermatology, Venereology and Allergology, Faculty of Medicine, Medical University of Gdansk, 80-214 Gdańsk, Poland

**Keywords:** Interleukin 35, atopic dermatitis, regulatory T cell, regulatory B cell, iTr35 cells, immunological disturbances

## Abstract

Interleukin 35 (IL-35), a new member of the IL-12 family of heterodimeric cytokines, could induce two different types of regulatory cells including regulatory T and B cells such as IL-35-induced regulatory T cells and IL-10-producing regulatory B cells (IL-10+Bregs), and IL-35-producing regulatory B cells (IL-35+Bregs). These cells appear to play an important role in modulating the immune system in numerous diseases. Several findings suggested that the expression of IL-35 is dysregulated in many autoimmune, inflammatory, and allergic diseases. Due to the functions of IL-35, it seems that this cytokine may act as an efficient therapeutic strategy for numerous conditions including atopic dermatitis (AD). We aimed to provide a comprehensive overview of the role of IL-35 in modulating the immune system. Additionally, we highlight IL-35 as a specific immunological target, discuss its possible involvement in the pathogenesis of AD, and hypothesize that IL-35 may become a novel target for the treatment of AD. However, further studies are required to evaluate this hypothesis.

## 1. Introduction

Interleukin 35 (IL-35) represents a new member of the IL-12 family of heterodimeric cytokines consisting of an α-subunit and a β-subunit, which also contains IL-12, IL-23, and IL-27 [1]. IL-35 is made up of the IL-12α chain p35 and the IL-27β chain Epstein–Barr virus induced 3 (EBI3) [2]. The expression of p35 and EBI3 subunits may be up-regulated under the influence of pro-inflammatory stimuli such as TNF-α, IFN-γ, TLR3, and TLR4 ligands in different cell types [3]. The secretion pattern of the IL-35 distinguishes it from the other members of the IL-12 family, which are primarily secreted by antigen-presenting cells (APCs) [4]. It is believed that the main source of IL-35 are regulatory T cells developing in the thymus (CD4+CD25+Foxp3+ T cells), named natural regulatory T cells (nTregs). Moreover, in contrast to mouse nTregs, which constitutively express IL-35, human nTregs only produce IL-35 after stimulation [5,6]. Collison et al. showed that the production of IL-35 increased following contact regulatory T cells (Tregs) with conventional T cells (Tconv). It was also suggested that IL-10 and IL-35 act together for maximal suppression mediated by Tregs and that their function is enhanced by Tconv cells [7]. IL-35 induces the unique population of the peripheral induced regulatory cells producing additional amounts of IL-35. The generation of these cells is named IL-35-producing regulatory T cells(iTr35) and does not express FoxP3 [8]. In addition, it was proved that IL-35 induces the conversion of regulatory B cells (Bregs) to the Breg subset that produces IL-35 as well as IL-10. Thus, regulatory B cells are also a source of IL-35 [9,10]. Dendritic cells, vascular endothelial cells, smooth muscle cells, and monocytes were also shown to secrete IL-35 by stimulations of various proinflammatory cytokines and bacterial endotoxin LPS [11]. Additionally, some cancer cells appear to produce IL-35 [12]. In addition to the secretion pattern, IL-35 differs from other family members in the fact that it is considered a strict immunosuppressive/anti-inflammatory cytokine. It was shown that trophoblast cells constitutively secrete IL-35 during pregnancy, which has a crucial role in preserving maternal–fetal tolerance [13,14]. These results support the immunosuppressive capability of this cytokine. Interestingly, unlike transforming growth factor-β (TGF-β), which is constitutively expressed, IL-35 is suggested to be expressed in human tissues in response to inflammation [11]. However, a recent study on mice models has revealed that IL-35 is constitutively produced by cells in the retina, including the photoreceptor rod cells and the cone cells, which indicate that its housekeeping functions maintain ocular immune privilege [15].

In this review, we focus on the current understanding of IL-35, including its biological function in regulating the immune system as well as in different diseases. In addition, we highlight IL-35 as a specific immunological target and discuss its possible involvement in the pathogenesis of atopic dermatitis (AD) and its possible relevance in the context of AD therapy. We hypothesize that IL-35 may become a novel target for the treatment of AD. From this work, we also wanted to open new insights into the pathogenesis of AD.

## 2. Receptor and Signaling Pathway of IL-35

IL-35 is unique from other members of the IL-12 family in its receptor. The IL-35 receptor may be a heterodimer or homodimer consisting of the configuration of the gp130, IL-12Rβ2 chain, and IL-27Rα, which activates signaling pathways dependent on the proteins STAT. Potential configurations of IL-35 receptors are gp130-gp130, IL-12Rβ2-IL-12Rβ2, IL-12Rβ2-gp130, and IL-12Rβ2-IL-27Rα [16].

IL-12Rβ2 is expressed on the surface of activated T cells, NK cells, B cells, and dendritic cells [15,16,17,18]. The IL-12Rβ2 subunit is undetectable on naive resting CD4+ T cells but its expression may be significantly altered by different molecules. It was shown that IL-2 and IL-27 are important inducers of the IL-35 receptor. There is evidence that these cytokines increase the expression of the IL-12β2 receptor chain on T cells, and thus, increase sensitivity to IL-35-mediated suppression [16]. In previous studies, IFN-γ was found to up-regulate the expression of the IL-12β2 subunit, while IL-4 inhibits the expression of this subunit [17]. IL-12 and TNF-α were also described as molecules up-regulating the IL-12β2 subunit [16].

IL-27Rα is mainly expressed by activated CD8+ T cells, CD4+ T cells, B cells, monocytes, and gp130 expressed by most immune cells [18,19].

**Figure 1:**
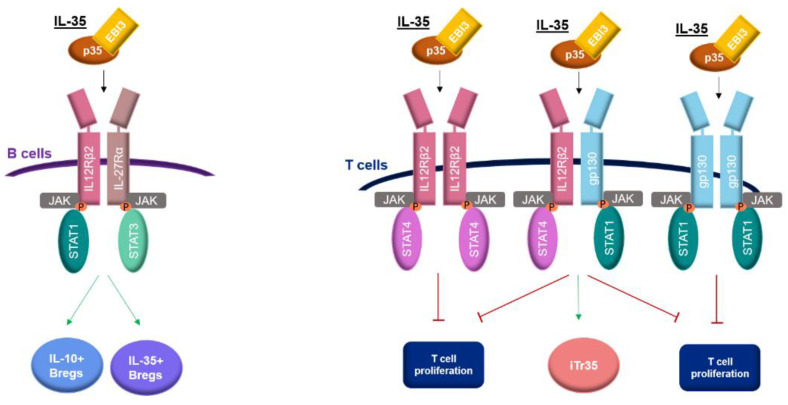
Receptor and signaling pathway of IL-35. IL-35 (Interleukin 35), IL-12Rβ2 (Interleukin 12 receptor subunit beta 2), IL-27Rα (Interleukin 27 receptor subunit alpha), JAK (Janus kinase), P (Phosphorus), STAT1 (Signal transducer and activator of transcription 1), STAT3 (Signal transducer and activator of transcription 3), IL-10+Bregs (IL-10-producing regulatory B cells), IL-35+Bregs (IL-35-producing regulatory B cells, STAT4 (Signal transducer and activator of transcription 4), gp130 (Glycoprotein 130), iTr35 (IL-35-producing T cells). In B cells, IL-35 signals through IL-12Rβ2-IL-27Rα and promotes the phosphorylation of STAT1 and STAT3, thereby inducing the generation of IL-35+Bregs and IL-10+Bregs. In T cells, IL-35 signaling through IL-12Rβ2-IL-12Rβ2 and gp130-gp130 leads to the phosphorylation of STAT4 and STAT1, respectively, resulting only in T cell suppression. IL-35 signal transduction via IL-12Rβ2-gp130, which activates STAT 4 and STAT1, can mediate the T cell suppression and induction of iTr35. The complete receptor of IL-12Rβ2-gp130 is required for the generation of iTr35. The green arrows indicate induction effects, and the red T-shaped ends indicate suppression effects.

The IL-35 signaling pathway, consisting of JAK1, JAK2, STAT1, STAT3, and STAT4 molecules, is different in B cells and T cells [4]. In B cells, IL-35 signaling through IL-12Rβ2-IL-27Rα leads to the phosphorylation of STAT1 and STAT3, resulting in the production of two population regulatory B cells that secrete IL-35 (IL-35+Bregs) and IL-10 (IL-10+Bregs) [10]. In T cells, IL-35 activates STAT1 and STAT4 via homodimeric receptors including IL-12Rβ2-IL-12Rβ2 and gp130-gp130, and one heterodimeric receptor IL-12Rβ2-gp130. The two homodimeric receptors are associated with only suppressing T cell proliferation while via heterodimeric receptor IL-35 mediates the suppression of T cell proliferation and conversion of T cells into IL-35-induced regulatory T cells (iTr35). However, both the complete heterodimeric receptor (IL-12Rβ2-gp130) and STAT1 and STAT4 signaling are required for this conversion [16] (Figure 1).

## 3. Expression of IL-35 in Human Diseases

Research findings suggested that the expression of IL-35 is dysregulated in many diseases. Moreover, in different diseases, IL-35 may play different roles. In the majority of diseases, research findings indicate a protective role of IL-35 by inhibiting inflammation and immune modulation. However, there are also some conflicting data. For instance, some studies found upregulated IL-35 in active systemic lupus erythematosus (SLE) patients and in patients with treatment-naïve early rheumatoid arthritis (RA); levels of this cytokine significantly decreased after the administration of treatment, which suggests that IL-35 may be pro-inflammatory in some diseases [20,21]. On the other hand, the increased serum levels of IL-35 may be a response to severe inflammation as compensation feedback to inhibit severe inflammation. It has been described that the upregulating expression of IL-35 subunits follows proinflammatory stimuli [3].

Table 1 summarizes the findings about IL-35 levels and biological functions in some human diseases.

## 4. Biological Function of IL-35

It was described that IL-35 may block the development of Th1 cells by limiting early T-cell proliferation [2]. Liu et al. showed that IL-35 reduces the generation of Th1 cells while enhancing IL-10 production in acute graft-versus-host disease (aGVHD) [37]. Jiang et al., in addition to the inhibitory effect of IL-35 on the production of Th1 cytokines, demonstrated a suppressing function on CD8+ cell activity by the inhibiting expression of costimulatory molecule CD28 [38]. IL-35 has been shown to suppress GATA-3 expression, which is an important transcription factor necessary for the development of type 2 innate lymphoid cells (ILC2s) and Th2 cells [39,40] and IL-4 repression, resulting in the inhibition of type 2 cytokine production from naive CD4+ T cells [27]. Liu et al. reported that IL-35 inhibits the proliferation of ILC2s and the production of type II cytokine by ILC2s more effectively than IL-10 and TGF-β. In addition, the inhibition process occurred by up-regulating IL-12Rβ2 and gp130 by ILC2s after stimulation of IL-35, which suggests a direct regulatory role for IL-35 on ILC2s [41]. The study evaluating the effect of IL-35 on human nasal epithelial cells showed the inhibiting effect of this cytokine on the production of IL-25, IL-33, and TSLP [42]. Airway inflammation in the mice model was significantly reduced after local administration of a plasmid that enhances IL-35 production. Levels of eosinophilia, neutrophilia, total IgE, and the Th2 cytokine IL-4 were observed to be decreased [43]. Furthermore, it was reported that IL-35 may mediate the conversion of Th2 cells to the iTr35; however, IFNγ can inhibit this process [1]. Scientific reports indicate that IL-35 inhibits the differentiation of Th17 cells through the down-regulation of retinoid-related orphan receptor γt (RORγt), and retinoid-related orphan receptor α (RORα), which are essential for Th17 development and function, and thus, reduce IL-17 levels. EBI3, which associates with p35 to form IL-35, was found to negatively regulates the expression of IL-17, IL-22, and ROR γt, which is the key transcription factor regulating Th17 cell differentiation [44]. Okada et al. also found a direct inhibitory effect of IL-35 on Th17 differentiation via inhibiting the expression of RORα and RORγt mRNA in Th17 cells, suppressing IL-17 expression [45]. Very similar results were obtained by Yan et al. The authors showed that IL-35 treatment in patients with proliferative diabetic retinopathy reduced RORα, RORγt, and Th17 cells [46].

**Figure 2:**
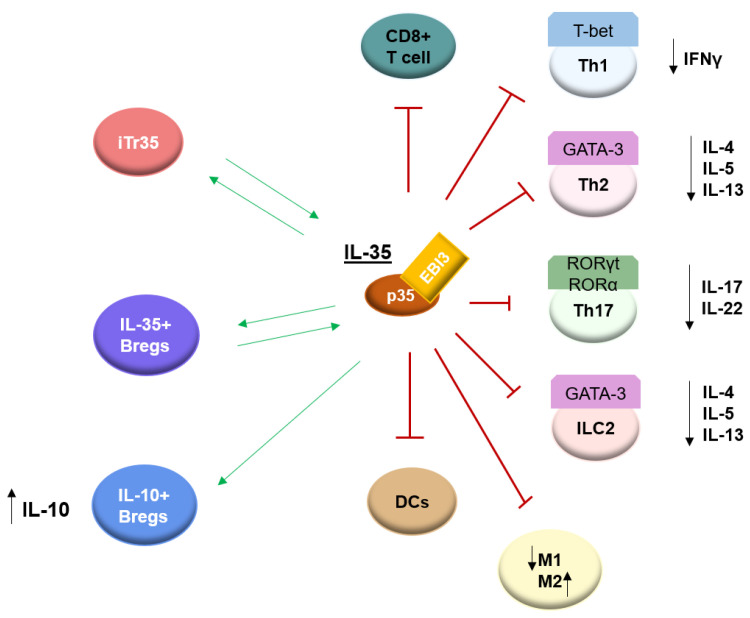
The function of IL-35. Th1 (T helper cells 1), INFγ (Interferon gamma), Th2 (T helper cells 2), IL-4 (Interleukin 4), IL-5 (Interleukin 5), IL-13 (Interleukin 13), Th17 (T helper 17), IL-17 (Interleukin 17), IL-22 (Interleukin 22), ILC2 (Innate lymphoid type-2-cells), DCs (Dendritic cells), M1 (M1 macrophages), M2 (M2 macrophages), IL-10+Bregs (IL-10-producing regulatory B cells), IL-35+Bregs (IL-35-producing regulatory B cells), iTr35 (IL-35-producing T cells). IL-35 can block the development of Th1, Th2, Th17, and ILC2 cells by suppressing transcription factors such as T-bet, GATA-3, RORα (retinoid-related orphan receptor α), and RORγt (retinoid-related orphan receptor γt), resulting in a decrease in corresponding cytokines. IL-35 can also suppress CD8+ T cells and DCs, decrease M1, and increase M2. IL-35 can induce iTr35 and IL-35+Bregs generation and the resulting cells produce additional IL-35. IL-35 can induce IL-10+Bregs which secrete IL-10. The green arrows indicate induction effects and the red T-shaped ends indicate suppression effects.

IL-35 also suppresses the differentiation and maturation of dendritic cells [47]. In addition, to investigate the effect of IL-35 on the M1/M2 ratio of macrophages, a study found that IL-35 treatment decreased the pro-inflammatory (M1) macrophages and increased anti-inflammatory (M2) macrophages in experimental type 1 diabetes [48]. In psoriasis, IL-35 was also described as a regulator of the M1/M2 macrophage ratio [49]. As previously mentioned, IL-35 can induce the unique population of the regulatory cells termed iTr35 and IL-10-producing regulatory B cells (IL-10+Bregs), and IL-35-producing regulatory B cells (IL-35+Bregs) [8,9,10] (Figure 2). The function of these regulatory cells will be discussed in the further part of this article.

Taking the above into account, it can be said that IL-35 is a cytokine playing a role in modulating an adaptive immune response and an innate immune response. Moreover, unlike many inflammatory autoimmune diseases where IL-35 appears to play a protective role, high levels of this cytokine have been linked to cancer onset. [3,50]. Additionally, IL-35 has been shown to contribute to the development of chronic infections. For instance, it has been demonstrated that IL-35 promotes the replication of the HBV virus [51].

## 5. IL-35-Induced Regulatory T Cells (iTr35)

The regulatory T cells are molecules that play an important role in modulating the immune system, maintaining tolerance to self-antigens, and preventing autoimmune disease [52]. They can be divided into two main subtypes: natural Tregs (CD4+CD25+Foxp3+ T cells), which develop in the thymus, and induced Tregs, which are derived from naïve CD4+ T cells in the periphery after TGF-β or IL-10 stimulation. TGF-β-induced regulatory T cells express Foxp3 and mainly secrete TGF-β. In turn, IL-10-induced regulatory T cells (Tr1) do not express Foxp3 and are known to the fact-producing IL-10 [53,54,55,56].

The population of iTr35 is the newest type of regulatory T cell and differs from the above-mentioned induced regulatory T cells. First, in contrast to TGF-β-induced regulatory T cells, iTr35 does not express FoxP [38]. Moreover, it was established that iTr35 mediates suppression via IL-35 but not via the other inhibitory cytokines IL-10 or TGF-β, differentiating them also from other previously known induced regulatory populations [8].

In patients with grass pollen allergy, iTr35 cells were found to be dysregulated and treatment with sublingual immunotherapy recovered these cells. The authors also found that IL-35 secreted by iTr35 has suppressed aberrant type 2 immune responses elicited by group 2 innate lymphoid cells (ILC2s) and Th2 cell proliferation and cytokine production [57]. Furthermore, Liu et al. found that iTr35 suppresses the differentiation of ILC2s and the expression of type cytokines by ILC2s through ICOS: ICOSL cell–cell contact and IL-35 [41]. Research on iTr35 in asthma has determined the decreased frequency of these cells and IL-35 levels in peripheral blood mononuclear cells (PBMCs) in patients with allergic asthma compared with asymptomatic (patients without Derp1 allergy) and healthy individuals. Additionally, it was shown in vitro that iTr35 cells inhibited effector T cells (Teff) proliferation, the naïve CD4+ T-cell proliferation and differentiation into Th2 cells, the GATA-3 mRNA expression level, and allergen-induced Th2 cytokine production in an IL-35-dependent manner, suggesting that allergen-specific iTr35 cells inhibit Th2-immune responses at the transcriptional and differentiation levels [27]. It has also described the ability of iTr35 to downregulate the development and differentiation of Th17 cells [58].

Summing up, these studies present iTr35 in light of the potential novel immune regulators, which could benefit AD patients. However, no study has measured the frequency of iTr35 in patients with the AD so far, and the role of this new subset of Tregs in patients with AD remains unknown.

## 6. Regulatory B Cells (Bregs)

Regulatory B lymphocytes (Bregs) participate in the maintenance and restoration of immune homeostasis by suppressing immune-mediated inflammation. Their role was described in several health conditions such as autoimmune diseases, cancer, infections, transplantation, pregnancy, and in allergic diseases [59].

As previously mentioned, IL-35 is capable of inducing IL-10-producing regulatory B cells (IL-10+Bregs) and IL-35-producing regulatory B cells (IL-35+Bregs) [10]. The most examined mechanism of Bregs action is the production of IL-10. It has been described that IL-10+Bregs inhibit Th1, Th17, and CD8+ T cell responses and convert naïve CD4+ T cells into regulatory T cell populations and IL-10-secreting type-1 regulatory CD4+ T cells (Tr1) [60]. Recently, IL-35+Bregs were described as a new type of immunity regulator. Mice lacking IL-35 production by B cells were associated with a disability to recover from the T cell-mediated demyelinating autoimmune disease experimental autoimmune encephalomyelitis (EAE) [9]. This study indicates that IL-35+Bregs are critical regulators of immunity during autoimmune diseases. On the other hand, IL-35+Bregs were found to be upregulated in neoplastic diseases. For instance, in patients with advanced gastric cancer, an elevated level of IL-35+Bregs was associated with the progression of the disease [61]. There is accumulating evidence that Bregs induce and maintain allergen tolerance. During allergen immunotherapy (AIT), Bregs by IL-10 were found to suppress T effector cells including Th2 responses, induce Tregs, inhibit dendritic cells (DCs) maturation, modulate Tfh cell responses, and induce the production of anti-inflammatory IgG4 antibodies [62].

## 7. Immunological Imbalance in Atopic Dermatitis

Depending on the chronicity of lesions in atopic dermatitis (AD), there are observed changes in the dominance of various types of cytokines. In the phase of acute lesions, in the skin, the accumulation of cytokines from the Th2 and Th22 axes and to a lesser extent, Th17, is observed. With disease chronicity, there are observed significant increases in Th1 cytokines with the intensification of Th2 and Th22 responses [63]. According to the IgE levels, AD can be divided into extrinsic and intrinsic AD. Patients with the classic extrinsic (80%) phenotype are characterized by traditional immune polarization towards Th2, elevated IgE levels, and eosinophilia. The normal level of IgE and greater immune polarization towards Th1 and Th17/Th22 are associated with intrinsic AD (20%) patients [63]. In adults, AD is the result of immune polarization mainly towards Th2 and Th22 cytokines in blood and the skin. Pediatric AD is characterized by the expression of Th2, Th22, and Th17 cytokines, and the expression of Th17 cytokines is higher than in adults [63]. Moreover, depending on ethnicity, Asian AD with greater Th17 and lower Th1 axis activation than European American AD—characterized by mainly Th2, Th22, and Th1 immune polarization—and African American AD with primarily Th2/Th22 axis activation, can be distinguished [63].

Thus, the dominance of various types of cytokines involved in AD development depends on the phase of the disease, age, and race of the patient. The causes of immune dysregulation in AD are not well-established yet. The immune system in AD is heterogeneous and complex. It is known that both innate and adaptive immunity are involved in the pathogenesis of the disease.

## 8. IL-35 as a Novel Regulatory Cytokine and Its Potential Involvement in Pathogenesis and Therapeutic Effects in Atopic Dermatitis

IL-35 shares a common subunit p35 as well as a receptor chain IL-12Rβ1 with IL-12, which is known to be involved in the pathogenesis of AD. The upregulated secretion of IL-12 is observed in the chronic lesions of AD and is responsible for stimulating the differentiation of Th0 cells into Th1 type, resulting in the switch to a Th1-type cytokine milieu associated with increased IFN-γ expression [64]. Moreover, the association between the IL12Rβ1 promoter polymorphisms and the increased risk of AD in Japanese subjects has been found [65]. Additionally, in the Korean population, the polymorphism of the IL-12Rβ1 gene was significantly associated with the AD phenotype, especially the allergic type of AD [66]. Thus, the possible involvement of IL-35 in the pathogenesis of AD could be concluded.

IL-35 has been described as an immunomodulator and may be involved in the pathogenesis of AD in several ways. It has an important role in blocking Th2 cells, which are the key to AD development [27,40,67]. Furthermore, the upregulated expression of IL-33, IL-25, and TSLP is observed in patients with AD, and IL-35 could cause the suppression of these cells, which in turn will also result in the inhibition of a Th2 immune response [42] p. 35, [68]. TSLP triggers the onset of the inflammatory cascade in AD and has been described as a master regulator of type 2 immune responses [69]. This proinflammatory cytokine appears to be a promising therapeutic target, and indeed, results of various studies show the association between the downregulation of TSLP expression and an alleviation of the symptoms of AD [70,71]. Several clinical studies are currently ongoing with TSLP as a therapeutic target [72]. Considering the roles of TSLP and its involvement in AD pathogenesis, it seems that patients with AD could benefit from the inhibition of TSLP by IL-35.

There is evidence that IL-35 has a direct inhibitory effect on ILC2s from which AD patients may also benefit [41]. ILC2s, which are the innate counterparts of Th2 cells, have been reported to be increased in AD skin lesions. These cells produce proinflammatory cytokines such as IL4, IL5, and IL13 and it has been found that low levels of ILC2s are associated with lower skin inflammation in AD mice models [73,74]. In addition, IL-35 has been found to suppress the development of Th1 cells, which together with Th2 and Th22, drive the chronic phase of AD [2,63].

The activity of IL-35 has been found to directly inhibit the differentiation of Th17 cells, which also indicates the possible involvement of this interleukin in the pathogenesis of AD [44,45,46]. IL-17 and IL-22 were described to contribute to skin barrier dysfunction [67]. Moreover, in murine AD models, IL-17 has been described to mediate Th2 immune responses, and IL-17 deficiency led to impaired Th2 induction [75]. Th17 response in AD as a therapeutic target is considered. This polarization is characteristic for children and Asian AD, who may be the targeted group considering personalized treatment according to immunotypes. Some phase II clinical trials have been carried out with a focus on targeting Th17 cytokines in the treatment of moderate-to-severe AD patients [76]. However, the key point with the interpretation of the results should be the fenoendotype characteristic of the studied group. Furthermore, it has been shown that IL-35 has a regulatory effect of IL-35 on the balance of Tregs/Th17 cells [77,78]. Some reports indicated an existing immune imbalance in Th17 and Tregs cells in AD, which may contribute to its pathogenesis and development [79]. Thus, the potential regulatory role of IL-35 in AD in this context can also be inferred.

**Figure 3:**
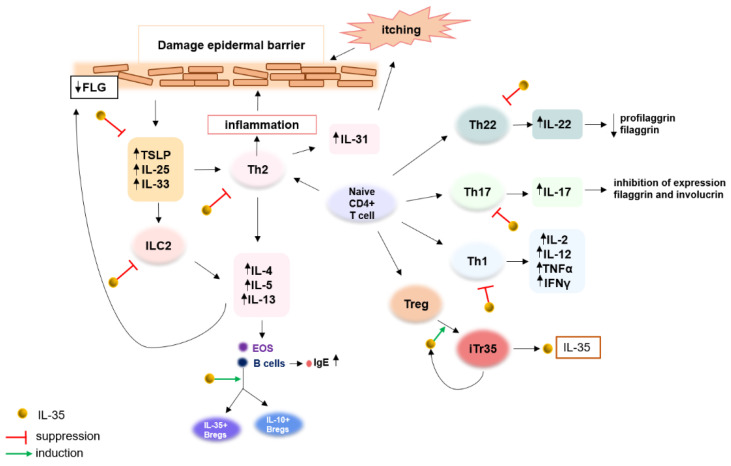
Potential involvement of IL-35 in the pathogenesis and its potential therapeutic effect in atopic dermatitis (AD). FLG (filaggrin), TSLP (Thymic stromal lymphopoietin), IL-25 (Interleukin 25), IL-33 (Interleukin 33), Th2 (T helper cells 2), ILC2 (Innate lymphoid type-2-cells), IL-4 (Interleukin 4), IL-5 (Interleukin 5), IL-13 (Interleukin 13), EOS (Eosinophils), IL-10+Bregs (IL-10-producing regulatory B cells), IL-35+Bregs (IL-35-producing regulatory B cells), IL-31 (Interleukin 31), Th22 (T helper cells 2), IL-22 (Interleukin 22), Th17 (T helper cells 17), IL-17 (Interleukin 17), Th1 (T helper cells 1), IL-2 (Interleukin 2), IL-12 (Interleukin 12), TNFα (tumor necrosis factor alfa), INFγ (Interferon gamma), Treg (Regulatory T cells), iTr35 (IL-35-producing T cells), IL-35 (Interleukin 35). This figure presents the main cytokines involved in AD pathogenesis, the links between them, and their roles, indicated by black arrows. IL-35 may have anti-inflammatory effects in AD via the possible inhibition of Th1, Th2, Th17, and Th22 cell responses. IL-35 can induce the conversion of B cells into regulatory B cells that produce IL-35 as well as IL-10. IL-35 can also induce iTr35 cell generation, which releases an additional amount of IL-35, creating a positive feedback loop. The green arrows indicate induction effects and the red T-shaped ends indicate suppression effects.

Taking this into account, IL-35, in theory, would contribute to restoring balance to the observed immune dysregulation in AD (Figure 3). Undoubtedly, further studies are required to investigate the precise effect and signaling pathway of IL-35 in AD.

## 9. Regulatory T Cells in Atopic Dermatitis

The role of regulatory T cells in the pathogenesis of AD is widely discussed. Studies examining Tregs frequencies in AD obtained conflicting results. A study by Verhagen et al. aimed to determine the presence and function of regulatory T cells in the skin of individuals with atopic dermatitis, revealing significantly expressed IL-10-secreting Tr1 cells. In turn, CD25+Foxp3+ Tregs were absent [80]. In another study, CD4+CD25+FOXP3+ Tregs in the peripheral blood were not significantly altered but increased numbers of FOXP3+ Tregs were detected in AD skin [81]. Fujimura et al. also showed the presence of FOXP3+ Tregs in AD lesion skin [82]. Roesner et al., who analyzed circulating Tregs frequencies of adult patients with AD, detected a positive correlation between these cells and disease severity [83]. Similar results were obtained by Ito et al., who also showed increased frequencies of FOXP3-positive CD4+CD25+ T cells in peripheral blood and their association with disease severity [84]. Another study additionally found higher frequencies of circulating Tregs, but moreover, the authors showed impaired iTregs generation in AD patients [85]. In contrast, a meta-analysis that included Chinese populations has revealed a decreased proportion of Tregs in the peripheral blood of patients with AD [86].

Nevertheless, reports indicate a potential involvement of Tregs in the pathogenesis of AD. Thus, the fact that Tregs are the main source of IL-35 indirectly suggests a possible role for IL-35 in the pathogenesis of AD. Moreover, it was revealed that the loss of one of the IL-35 receptor subunits, gp130 or IL-12Rβ2, could impact the conversion ability of iTr35, and thus, IL-35 production [16]. Interestingly, one study found mutations in IL-12Rβ2 in atopic individuals [87]. Additionally, Chen et al. showed that IL-12Rβ2 may be regulated by miR-151a, which is involved in the pathogenesis of AD [88]. It is also known that IL-4, which is one of the main players in AD, inhibits IL-12Rβ2 expression, leading to the loss of IL-12 signaling [17]. Thus, the question whether genetic defects and the immunological regulation of IL-12Rβ2 expression interferes with the conversion of iTr35 and, consequently, the production of IL-35 by them in AD individuals comes to light. Although, on the other hand, the deficiency of IL-35 can significantly reduce the regulatory activity of Tregs [2].

## 10. Regulatory B Cells in Atopic Dermatitis

Regulatory B cells are an interesting research area due to their immunomodulation functions in several conditions. Unfortunately, the number of studies on the role of these cells in AD is limited. The first scientific reports on the regulatory role of B cells in AD arose in 2015 when a decreased number of IL-10-producing B cells was demonstrated in an Atopic Dermatitis-Like Mouse Model. In addition, the results of this study suggested that IL-10-producing B cells have a defective regulatory function on IgE secretion [89]. Additionally, Yoshihara et al. showed that the frequency of IL-10-producing regulatory B cells is decreased in patients with AD. Moreover, an inverse correlation between these cells and disease severity was found [90].

The precise role of IL-10-producing regulatory B cells in AD is not yet fully understood. However, the current reports about the aberrant number of these cells in AD indirectly indicate the potential involvement of IL-35 in AD pathogenesis due to its capability of inducing IL-10-producing regulatory B cells.

## 11. Discussion

As discussed above, IL-35 can inhibit the development of Th1, Th2, and Th17 cells in the early stage of the proliferation of these cells while inducing the generation of the regulatory T cells releasing IL-35, termed iTr35, as well as the conversion of Breg cells to a Breg subset that produces IL-35 and IL-10 [8,9,10]. Through the inhibition of Th1, Th2, and Th17 cells, IL-35 induces anti-inflammatory effects. In addition, the generations of iTr35 cells and Breg cells promoted by IL-35 play also a significant role in the regulation of immune responses via the secretion of immunosuppressive cytokines. For this reason, IL-35 acts as an important role in modulating the immunity system.

Dysregulated levels of IL-35 are observed in many diseases including asthma, allergic rhinitis, CSU, IBD, psoriasis, cancers, viral diseases, and connective tissue diseases as well as AD; research data suggest that IL-35 may play different roles in different diseases [20,21,22,23,24,25,26,27,28,29,30,31,32,33,34,35,36]. For some diseases, the results of IL-35 concentrations obtained in different studies appear to be contradictory. Regarding asthma, some studies indicate elevated levels of IL-35 in patients compared to healthy people [22,23]. On the other hand, some authors obtained opposite results and one study showed no significant differences between healthy and asthmatic patients [24,25,26]. The possible reasons for these different results may be the heterogeneity of airway inflammation in asthma patients and the different subtypes of this disease. Another reason may be that some of these studies included only children, others included only adults and some included patients of all ages. Finally, in the mentioned research, different samples including plasma, serum, and peripheral blood mononuclear cells (PMBCs) were tested. Conflicting results were obtained regarding SLE patients. In one mentioned study in Table 1, adults with newly diagnosed SLE presented decreased levels of IL-35 compared to healthy people [34]. In turn, Qiu et al. showed that newly diagnosed SLE patients have higher IL-35 serum levels compared to healthy people, and after prednisone treatment, the serum levels of this cytokine decreased significantly [20]. Perhaps, in the study by Qiu et al., the group of patients presented upregulated levels of IL-35 in response to severe inflammation. This hypothesis is supported by the fact that the serum level of this cytokine decreased after treatment. This may partially explain the differences in results between these two studies. Likewise, in the case of RA, IL-35 levels significantly decreased after treatment [21].

Due to the anti-inflammatory effects of IL-35, this cytokine is under consideration to be a promising drug for the treatment of inflammatory diseases. Additionally, the regulatory cells, including iTr35 cells and IL-35+Bregs with immunosuppressive capacity, could be induced via recombinant IL-35 protein therapy [74]. The use of IL-35 recombinant protein in the treatment of acute colitis and psoriasis showed good therapeutic effects in mouse models [91]. In another study, mice treated with IL-35 were characterized by significantly alleviated lupus flare and nephritis, which was associated with the expansion of Tregs and IL-10-producing Bregs [92]. IL-35 appears to be a crucial cytokine able to regulate immune responses and can be a therapeutic target for a large variety of diseases. Although the levels and immunomodulatory effects of IL-35 may vary from disease to disease, this fact does not exclude this cytokine from being considered a therapeutic target. Targeting therapy to reduce or upregulate IL-35 concentration would be used depending on its concentration in a given disease. Considering the role of this interleukin in maintaining the immune balance, it seems to be involved in the pathogenesis of AD and could also be a promising new therapeutic target in this disease. However, this proposal has limitations. To date, only a few human studies have been carried out on the serum levels of IL-35 in AD [30,36]. Nevertheless, the results from these studies seem inconsistent. One study suggests a decreased serum concentration of IL-35 in AD, which may indicate the involvement of this cytokine in the pathogenesis of the disease. However, based only on this one research, it cannot be clearly stated. The serum level and function of IL-35 in AD still need to be investigated in further studies to confirm our hypothesis.

In recent years, great advances in new therapeutic strategies for the treatment of AD emerged: monoclonal antibodies directed against interleukins involved in disease pathogenesis or their receptors, and small molecule drugs inhibiting JAK-STAT signaling pathways have been developed. By their counteraction, these drugs focus mainly on suppressing components of the immune system involved in the processes underlying the inflammatory response in AD [93,94]. The presented role of IL-35 and its potential use in the treatment of a variety of diseases with similar immune polarization to AD opens a new potential perspective on the treatment of AD. Perhaps in the future, it is worth considering immunomodulatory therapies, which, unlike currently available therapies, will focus on stimulating molecules such as regulatory cells, which through their naturally secreted immunosuppressive cytokines, will modulate the immune system in AD, leading to the expected therapeutic goal.

## 12. Conclusions

Based on the mentioned data, IL-35 appears to have a significant influence on modulating the immune response in many inflammatory diseases. It might be that its activity depends on the cytokine milieu. Most of the available data indicate an anti-inflammatory and protective effect of this cytokine, but a few reports suggest its pro-inflammatory effect. Therefore, the function of IL-35 in the pathogenesis of many diseases including AD still needs to be investigated in the future. Further research will not only elucidate the role of IL-35 in AD pathogenesis, but may also identify a novel therapeutic target.

## Figures and Tables

**Table 1:** IL-35 levels in some human diseases.

Country and Authors	Patients and Condition	IL-35 Level Measurement Method	Level in Blood	Additional Information
Saudi Arabia 2022; Abushouk et al. [22]	Children with Asthma vs. Healthy individuals	ELISA; serum	Increased; Asthma patients: 28.06 ± 8.39 pg/mL; Healthy control: 5.38 ± 5.54 pg/mL	IL-35 levels negatively correlated with levels of IgE.
China 2015; Wong et al. [23]	Adults and children with Asthma vs. Healthy individuals	ELISA; plasma	Increased; Asthma patients: 55.9 (6.6–419.0) ng/mL; Healthy control: 2.5 (0.1–16.1) ng/mL	IL-35 levels positively correlated with disease severity scores.
China 2015; Wang et al. [24]	Adults with Asthma vs. Healthy individuals	ELISA; plasma; qPCR; IL-35 mRNA expression levels in PBMCs	Decreased levels of IL-35 in plasma *. Decreased mRNA levels of the IL-35 subunits *	IL-35 levels negatively correlated with the frequency of IL-4-producing CD8+ T (Tc2) cells and with the IL-4 level.
Iran 2017; Khoshkhui et al. [25]	Children with Asthma vs. Healthy individuals	ELISA; serum	No significant difference; Asthma patients: 30.9 (3.8–110.7) pg/mL; Healthy control: 30.2 (6.1–239.7) pg/mL	
China 2014; Ma et al. [26]	Children with Asthma vs. Healthy individuals	ELISA; serum; qPCR; IL-35 mRNA expression levels in PBMCs	Decreased levels of IL-35 in serum *. Decreased mRNA levels of the IL-35 *	IL-35 levels negatively correlated with IL-4 levels and positively with IFN-y levels.
China 2020; Wang et al. [27]	Adults: Allergic asthmatic patients vs. Asymptomatic sensitized patients vs. Healthy individuals	Flow cytometry; the iTr35 cell frequency in PBMCs	Decreased iTr35 cell frequencies and IL-35 levels in allergic asthmatic patients *	1. sIgE levels negatively correlated with the percentage of iTr35 cells in asthmatic individuals; 2. Th2 cytokines levels negatively correlated with the iTr35 cell frequency in asthmatic, asymptomatic, and healthy individuals; 3. Th2 cytokines and sIgE levels negatively correlated with IL-35 levels in asthmatic patients.
China 2020; Xie et al. [28]	Children with Allergic rhinitis vs. Healthy individuals	ELISA; serum	Decreased *	IL-35 levels negatively correlated with IL-17 and IL-23 levels.
China 2021; Huang et al. [29]	Children with Allergic rhinitis vs. Healthy individuals	ELISA; plasma	Decreased; Allergic rhinitis patients: 138.52 ± 50.13 ng/mL; Healthy control: 426.45 ± 80.15 ng/mL	IL-35 levels negatively correlated with ILC2s.
China 2018; Chen et al. [30]	CSU patients vs. AD patients vs. Healthy individuals	ELISA; serum	Decreased in CSU patients: 73.46 ± 9.146 ng/mL; AD: 1264 ± 186.9 ng/mL; Healthy control: 1349 ± 170.7 ng/mL	
China 2014; Li et al. [31]	Patients with IBD: ulcerative colitis (UC) Crohn’s disease (CD) vs. Healthy individuals	ELISA; serum	Decreased in UC and CD *.	IL-35 levels negatively correlated with UC activity.
China 2018; Li et al. [32]	Adults with Psoriasis vs. Healthy individuals	ELISA; plasma; qPCR; PBMCs; Ebi3 and p35 mRNA levels	Decreased IL-35 levels: 2.67 (1.38–21.81) ng/mL vs. 7.92 (2.88–41.07) ng/mL; Decreased mRNA levels of EBI3 and p35 *	IL-35 levels negatively correlated with IFNy, TNF-a, levels of IL-23, -17, and -22, or the PASI and positively with TGF-β and IL-10 levels.
Egypt 2022; Elbana et al. [33]	Adults with Psoriasis vs. Healthy individuals	ELISA; serum	Decreased; Psoriasis patients: 72.65 ± 16.24 ng/L; Healthy control: 451.02 ± 117.16 ng/L	IL-35 levels negatively correlated with TNF-α, IL-17, IFN-γ.
China 2019; Ye et al. [34]	Adults with newly diagnosed SLE vs. Healthy individuals	ELISA; plasma	Decreased *	IL-35+B cells and IL-10+B cells decreased; The percentage of IL-35+Bregs and IL-35 levels inversely correlated with the SLE disease activity index.
China 2013; Qiu et al. [20]	Patients with newly diagnosed SLE; Pre-treatment SLE vs. post-treatment SLE vs. Healthy individuals	ELISA; serum	Increased; Pre-treatment: 9.94 (4.28–63.83) ng/L; Post-treatment: 5.78 (4.08–54.95) ng/L; Healthy control: 4.74 (3.38–12.45) ng/L	After prednisone treatment, the serum levels of IL-35 decreased significantly.
China 2019; Li et al. [35]	Adults with RA vs. Healthy individuals	ELISA; serum	Increased; RA patients: 6.3 (4.8–10.0) pg/mL; Healthy control: 1.3 (0.7–2.5) pg/mL	IL-35 levels negatively correlated with diseases activity based on ESR (DAS28-ESR).
Czech Republic 2015; Šenolt et al. [21]	Adults with treatment-naïve early RA vs. established RA vs. Control patients with osteoarthritis	ELISA; Serum and synovial fluid	Serum: Increased in patients with treatment-naïve early RA at baseline: 81.6 (20.7–564.4) pg/mL; Control patients: 10.4 (0.6–64.1); Established RA 22.8 (1.2–145.5) pg/mL; Synovial fluid: Increased Established RA: 445.0 (40.7–1908.0) pg/mL; Control patients: 125.5 (39.1–1062.0) pg/mL	IL-35 levels significantly decreased after treatment initiation to 36.5 (5.0–204.8) pg/mL; Synovial fluid IL-35 levels positively correlated with disease activity assessed by CRP and DAS28.
Egypt 2022; Kiwan et al. [36]	Patients with AD vs. Healthy individuals	ELISA; serum	Decreased; AD patients: 69.7 ± 14.8 ng/L; Healthy control: 415.96 ± 99.25 ng/L	IL-35 levels negatively correlated with diseases severity assessed by SCORAD, TNF- α, IL-17 and positively correlated with TGF-β.

Abbreviations: ELISA: enzyme-linked immunosorbent assay; qPCR: quantitative polymerase chain reaction; PBMCs: peripheral blood mononuclear cells; sIgE: specific IgE; ILC2s: innate lymphoid type-2 cells; CSU: chronic spontaneous urticaria; AD: atopic dermatitis; IBD: inflammatory bowel disease; SLE: systemic lupus erythematosus; RA: rheumatoid arthritis; * Some authors presented the results on diagrams, making it difficult to read the exact numerical values.

## Data Availability

The data presented in this study are available in the PubMed database—https://pubmed.ncbi.nlm.nih.gov/ or under the links cited of cited websites.
